# Nutritional and Hydration Status and Adherence to Dietary Recommendations in Dalmatian Dialysis Patients

**DOI:** 10.3390/nu14173553

**Published:** 2022-08-29

**Authors:** Ela Kolak, Josipa Radić, Marijana Vučković, Dora Bučan Nenadić, Mirna Begović, Mislav Radić

**Affiliations:** 1 Department of Nutrition and Dietetics, University Hospital Centre Split, 21000 Split, Croatia; 2 Department of Nephrology and Dialysis, University Hospital Centre Split, 21000 Split, Croatia; 3 Department of Internal Medicine, School of Medicine, University of Split, 21000 Split, Croatia; 4 Student of School of Medicine, University of Split, 21000 Split, Croatia; 5 Department of Clinical Immunology and Rheumatology, University Hospital Centre Split, 21000 Split, Croatia

**Keywords:** hemodialysis, peritoneal dialysis, nutritional status, dietary intake, dietary recommendations, nutrient adequacy, malnutrition inflammation score

## Abstract

Protein-energy wasting (PEW) is considered one of the major complications of chronic kidney disease (CKD), particularly in dialysis patients. Insufficient energy and protein intake, together with clinical complications, may contribute to the onset and severity of PEW. Therefore, the aim of the study was to analyze the differences in nutritional and hydration status and dietary intake among Dalmatian dialysis patients. Fifty-five hemodialysis (HD) and twenty peritoneal dialysis (PD) participants were included. For each study participant, data about body composition, anthropometric, laboratory, and clinical parameters were obtained. The Malnutrition Inflammation Score (MIS) and two separate 24-h dietary recalls were used to assess nutritional status and dietary intake. The Nutrient Adequacy Ratio (NAR) and Mean Adequacy Ratio (MAR) were calculated to compare actual dietary intake with recommended intake. Additionally, the estimated 10-year survival was calculated using the Charlson Comorbidity Index. The prevalence of malnutrition according to MIS was 47.3% in HD and 45% in PD participants. Significant differences in fat tissue parameters were found between HD and PD participants, whereas significant differences in hydration status and muscle mass parameters were not found. A significant difference in NAR between HD and PD participants was noticed for potassium and phosphorus intake, but not for MAR. MIS correlated negatively with anthropometric parameters, fat mass, visceral fat level and trunk fat mass, and iron and uric acid in HD participants, whereas no significant correlations were found in PD participants. The estimated 10-year survival correlated with several parameters of nutritional status in HD and PD participants, as well as nutrient intake in HD participants. These results indicate a high prevalence of malnutrition and inadequate dietary intake in the Dalmatian dialysis population which, furthermore, highlights the urgent need for individualized and structural nutritional support.

## 1. Introduction

A gradual deterioration of nutritional status has been observed in patients approaching end-stage renal disease and those undergoing maintenance dialysis, resulting in a catabolic state and fat and muscle tissue wasting [1,2]. Protein-energy wasting (PEW), defined as the depletion of body protein and energy reserves associated with chronic kidney disease (CKD) [3], is regarded as one of the most serious and important complications of CKD, especially for patients undergoing dialysis [4,5]. A combination of insufficient energy and protein intake, uremia-induced alterations, metabolic acidosis, inflammation, nutrient loss, gastroenterological distress due to the use of phosphorus binders and iron supplements, depression, lack of physical activity, and frailty contribute to PEW onset and its severity [6,7]. In addition, other factors, including dialysis-specific catabolism and nutrient loss, early satiety associated with mandatory peritoneal glucose absorption, and low socioeconomic status, may influence PEW [7,8]. 

Among interventions for reducing the disease progression and its complications, there is a growing emphasis on lifestyle and dietary changes depending on the stage of CKD and renal replacement therapy (peritoneal dialysis (PD), hemodialysis (HD), or kidney transplantation) [9]. The goals of nutritional management in CKD include not only preserving kidney function, but also maintaining optimal nutritional status, primarily by preventing PEW, electrolyte imbalances, and bone and mineral abnormalities, as well as improving the quality of life and patient-related disease outcomes [10,11,12].

Dietary recommendations for patients undergoing dialysis comprise high energy and protein intake, as well as careful management of the intake of fluid and selected micronutrients, such as phosphorus, potassium, sodium, and calcium [2,13]. The overlap in food rich in protein and the above-mentioned micronutrients forms an eating pattern that is difficult to adhere to, and which can lead to decreased energy and micronutrient intake. The adherence to such restrictions can contribute to a reduced intake of foods considered healthy, such as whole grains, vegetables, legumes, fruits, and nuts [14]. Although these recommendations may be necessary to prevent hyperkalemia, hyperphosphatemia, and metabolic acidosis [2], nutritional therapy in CKD is considered one of the most restrictive and challenging diets in all chronic diseases [10]. 

According to the new Kidney Disease: Improving Global Outcomes (KDIGO) guidelines, bi-annual nutritional screening should be considered for adults with CKD 3-5D, whereas nutritional assessment should be conducted within the first 90 days of dialysis initiation, annually or as indicated otherwise [15]. Furthermore, the most efficient nutritional interventions as suggested by the Modification of Diet in Renal Disease study are based on a structured dietary approach with frequent feedback during follow-up, patient education, and dietary interventions as needed [16]. Nutritional counselling and compliance to the dietary recommendations received could improve the nutritional status in patients treated with HD and PD, and could also allow an increased consumption of high-protein foods without exceeding optimal phosphorus, sodium, and potassium intake [2,17]. 

An alarmingly high rate of PEW has been observed among adults with CKD, with a PEW prevalence of 11 to 54% in 3-5D patients, and 28 to 52% in kidney transplant recipients [4]. Furthermore, compliance with the CKD-specific dietary recommendations in both HD and PD patients is not in line with current recommendations [18,19]. Considering that an adequate dietary pattern can improve nutritional status and quality of life, as well as reduce morbidity and mortality in dialysis patients, the aim of this study was to analyze nutritional and hydration status and dietary intake in Dalmatian patients treated with PD and HD, and to determine adherence to the dietary recommendations specific for this population of patients. 

## 2. Materials and Methods

### 2.1. Study Design and Population

This research, designed as a cross-sectional study, was carried out at the Outpatient Clinic for Clinical Nutrition, Division of Nephrology and Dialysis, Department of Internal Medicine, University Hospital Centre Split, Croatia, in the period between February and April 2022. Fifty-five (55) participants undergoing 4-hour HD treatment three times per week were recruited prior to a mid-week HD session, and twenty (20) participants undergoing PD were recruited during a regular visit to the nephrologist. Participants were subject to the following exclusion criteria: participants that changed dialysis modality; immobility; implanted pacemaker or cardioverter-defibrillator; stents or limb amputation; existing acute infection; existing active underlying malignant disease; existing oedema; corticosteroids intake; COVID-19 recovery or vaccination less than two months prior; cognitive impairment preventing completion of questionnaires; refusal to participate in the study. All participants were informed of the purpose of the study and gave written and verbal consent.

### 2.2. Body Composition and Anthropometric Measurements

Body composition was assessed for each study participant using the MC-780 Multi Frequency Segmental Body Mass Analyzer (Tanita, Tokyo, Japan). The scale sends a constant high-frequency current through the body and uses eight electrodes to measure the resistance of the various body tissues. This technology, known as bioelectrical impedance analysis, is used to assess body mass (kg), total body water (TBW; kg), extracellular water (ECW; kg), intracellular water (ICW; kg), muscle mass percentage (%), fat-free mass (kg), fat mass (kg and %), visceral fat, skeletal muscle index (SMI), trunk fat mass (kg and %), and phase angle (°). All participants were advised beforehand to follow the instructions from the device manual: to empty the bladder if residual kidney function was present, not to take any food or liquid for at least 3 h before the measurement, and to refrain from strenuous physical activity and alcohol consumption for least one day before the measurement [20]. In addition, body composition measurement for PD participants was conducted following PD fluid drainage, as recommended by KDIGO guidelines [15]. 

Just before measuring body composition, a stadiometer was used to determine the body height of the participants, and the body mass index (BMI) was calculated. Mid-upper arm circumference (MUAC), hip circumference (HC), and waist circumference (WC) were measured using non-stretchable, flexible body-measuring tape according to the instructions from the anthropometric standardization reference manual [21]. Handgrip strength (HGS) was measured on the hand without vascular access in HD participants, and alternating hands in PD participants using a hydraulic hand dynamometer (SAEHAN Corporation, Changwon, Korea). Three measurements were performed, and the average value was regarded as HGS.

### 2.3. Lifestyle Questionnaire, 24-h Dietary Recall, and Nutrient Adequacy Ratio

A lifestyle questionnaire that included questions about socio-demographic characteristics, as well as dietary habits and oral nutritional supplement intake, was obtained for each study participant. For dietary assessment, each study participant also completed two separated 24-h dietary recalls for non-dialysis days. The picture book was used for the estimation of food portion sizes, and converted to grams according to the instructions from A Users Guide to the Photographic Atlas [22]. For PD participants, additional energy intake from peritoneal dialysate was applied. Nutritional intake was analyzed using the software package and computer program, “Dietitian” ver. 206.0.000.

The Nutrient Adequacy Ratio (NAR) was estimated for fourteen nutrients (energy; protein; sodium; potassium; calcium; phosphorus; vitamins C, D, B6, and B12; thiamine; riboflavin; niacin; folic acid). The NAR for the energy and protein, as well as the above-mentioned minerals, was calculated as the actual intake of the nutrient divided by the recommended intake for HD and PD participants [15,23], whereas the NAR calculation for vitamins in both groups of participants was based on the recommended dietary intake for a healthy European population [24]. The Mean Adequacy Ratio (MAR) was estimated as a sum of the NAR values divided by the number of nutrients. Each NAR value was limited to not exceed 1.0. For both NAR and MAR, a value lower than 1.0 indicates intake lower than recommended for one or more nutrients, whereas a value of 1.0 indicates that actual intakes are consistent with the recommendations [25].

### 2.4. Malnutrition Inflammation Score

To assess the nutritional status of the participants, a Malnutrition Inflammation Score (MIS) specific for patients diagnosed with CKD was used. The score mentioned above consists of ten components that include nutritional history, physical examination, BMI, and laboratory values. The first seven components were taken from the original Subjective Global Assessment (SGA) questionnaire, and are related to weight change, dietary intake, gastrointestinal symptoms, functional capacity, comorbid conditions, and an assessment of the subcutaneous body fat and muscle wasting. The remaining three components are characteristic of MIS, and include data on BMI, serum albumin, and total iron-binding capacity (TIBC). Each component can be assigned a value from 0 to 3, whereas the sum of all component values can vary between 0 and 30, with a higher value indicating a more severe level of malnutrition, and a cut-off point set at >6 [26].

### 2.5. Medical History, Clinical and Laboratory Parameters

For each study participant, data about the duration of dialysis, presence of comorbid conditions, and data related to oral nutritional supplementation (ONS) such as prescription, as well as use and adherence to the prescription, were collected.

Regarding laboratory parameters, blood samples in fasting conditions were taken before a mid-week HD session for HD participants, and during a regular visit to a nephrologist for PD participants. Obtained data included concentrations of serum hemoglobin (Hb; g/L), mean corpuscular volume (MCV; fL), serum albumin (g/L), fasting blood glucose (FBG; mmol/L), uric acid (µmol/L), total cholesterol (mmol/L), low-density lipoprotein cholesterol (LDL; mmol/L), high-density lipoprotein cholesterol (HDL; mmol/L), triglycerides (mmol/L), sodium (mmol/L), potassium (mmol/L), phosphates (mmol/L), calcium (mmol/L), chloride (mmol/L), magnesium (mmol/L), total iron-binding capacity (TIBC; µmol/L), *C*-reactive protein (CRP; mg/L), and intact parathyroid hormone (iPTH; pmol/L).

iPTH was measured by an immunoassay analyzer (Cobas e601, Roche Diagnostics, Penzberg, Germany).

### 2.6. Charlson Comorbidity Index (CCI)

Charlson Comorbidity Index (CCI) is a validated, simple, and easily applicable method used for the prediction of 10-year survival in patients with multiple comorbidities. The score consists of 16 variables regarding the presence of disease, and is scored depending on the severity of the disease [27,28]. One point is awarded for myocardial infarction, congestive heart failure, peripheral vascular disease, cerebrovascular accident or transient ischemic attack, dementia, chronic obstructive pulmonary disease, connective tissue disease, peptic ulcer disease, mild liver disease, and uncomplicated diabetes; two points are awarded for hemiplegia, moderate to severe chronic kidney disease, diabetes with end-organ damage, localized solid tumor, leukemia, and lymphoma; three points are awarded for moderate-to-severe liver disease; and six points are awarded for metastatic solid tumor and AIDS. Additionally, one point is awarded for every decade age 50 years and over, with a maximum of four points [29]. A higher score indicates a higher severity of disease and mortality rate. The estimated 10-year survival was calculated using the following formula, 0.983^(eCCI × 0.9)^, where CCI is the Charlson Comorbidity Index.

### 2.7. Statistical Analysis

Categorical data are represented by absolute and relative frequencies. Differences of categorical variables were tested by a Chi-squared Test. The normality of the distribution of numerical variables was tested by the Shapiro–Wilk test. Numerical data were described by the median and the limits of the interquartile range. The differences between two independent groups were tested by Mann–Whitney’s U test. The correlation between numeric variables was evaluated by Spearman’s Correlation Coefficient ρ (rho). The level of significance was set at an Alpha of 0.05. The statistical analysis was performed using MedCalc^®^ Statistical Software, version 20.111 (MedCalc Software Ltd., Ostend, Belgium; Available online: https://www.medcalc.org (accessed on 7 June 2022)) [30].

## 3. Results

The study sample comprised fifty-five (55) HD participants, of whom, 30.9% (17) were women, and twenty (20) PD participants, of whom, 45% (9) were women. The median dialysis vintage was 47 months (interquartile range, IQR: 22–77 months) for HD participants, and 24 months (IQR: 8.5–36 months) for PD participants. The data about basic characteristics, body composition, and anthropometric parameters for each dialysis modality and the differences between them are shown in Table 1. Participants treated with HD were significantly older (*p* = 0.002), had higher dialysis vintage (*p* = 0.001), had a higher prevalence of type 2 diabetes mellitus (*p* = 0.03) and malignant diseases (*p* = 0.003), as well as a higher CCI score (*p* < 0.001). The estimated 10-year survival, on average, was 2% for HD participants, and 53% for PD participants. Regarding BMI, PD participants were overweight, whereas HD participants had normal body weight, but the mentioned difference did not reach a significant level. According to body composition analysis, HD participants had a significantly lower fat mass (%, *p* = 0.02; kg, *p* = 0.01), as well as trunk fat mass (%, *p* = 0.01; kg, *p* = 0.02) and handgrip strength values (*p* = 0.01), in comparison to the PD participants. No significant differences regarding hydration parameters were noticed between HD and PD participants.

The data about the biochemical parameters and differences among the two groups of participants observed are shown in Table 2. Significantly higher Hb (*p* = 0.03), FBG (*p* = 0.04), serum albumin (*p* < 0.001), and potassium (*p* < 0.001) values were noticed in HD participants, whereas PD participants had higher values of total cholesterol (*p* = 0.04) and LDL (*p* = 0.04).

The data obtained through the lifestyle questionnaire are presented in Table S1. A significant difference was noticed in employment status, where most of the HD participants were retired (*p* < 0.001). A lack of appetite was reported by 10 (18%) HD participants and one (5%) PD participant, whereas 15 (27%) HD participants and one (5%) PD participant had nausea. Furthermore, 44% of HD and 45% of PD participants had been prescribed oral nutritional supplements, but only 71% (of those prescribed) of HD and 56% of PD participants were using them according to the specialist’s recommendations, considering the frequency of intake and the intake itself.

**Figure 1:**
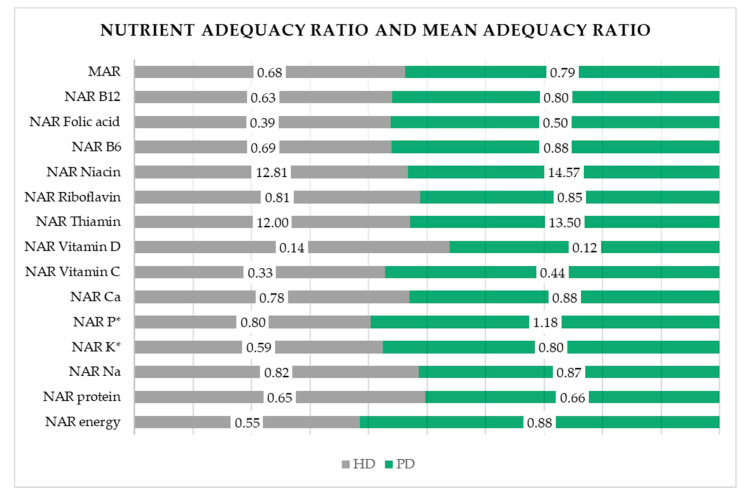
Nutrient adequacy ratios and mean adequacy ratio according to dialysis modality. * depicts statistically significant difference. *p*-values were obtained with the Chi-squared test (*p* < 0.05). Abbreviations: MAR—mean adequacy ratio, NAR—nutrient adequacy ratio, Ca—calcium, P—phosphorus, K—potassium, Na—sodium.

According to the 24-h dietary recalls, a significant difference, as shown in Table 3, was determined for the following nutrients: energy intake (*p* < 0.001), fat (*p* = 0.008), SFA (*p* = 0.02), dietary fiber (*p* = 0.04), vitamin E (*p* = 0.05), magnesium (*p* = 0.02), phosphorus (*p* = 0.04), copper (*p* = 0.005), and potassium (*p* = 0.02), with higher intakes noticed in PD participants. NAR values are graphically demonstrated in Figure 1, and detailed numerical data are shown in Table S2. The median of the MAR for HD and PD participants was 0.68 and 0.79, respectively, indicating a lower intake for several micronutrients than recommended in both groups of participants. A higher than recommended intake was noticed in HD participants for niacin and thiamin, whereas PD participants had a higher than the recommended intake for niacin, thiamine, and phosphorus. A significant difference in the NAR between the HD and PD participants was noticed for potassium (*p* = 0.01) and phosphorus (*p* < 0.001).

MIS values lower than 6 had 52.7% (29) HD and 55% (11) PD participants. The median MIS value was 5 (4–8) for HD and 5 (2–7) for PD participants without statistically significant difference regarding the dialysis modality as shown in Table 4. Significantly higher serum albumin (*p* < 0.001) and lower TIBC (*p* = 0.006) values were found for HD participants. Moreover, participants undergoing HD spent significantly more years on dialysis treatment (*p* = 0.01) than PD participants.

**Figure 2:**
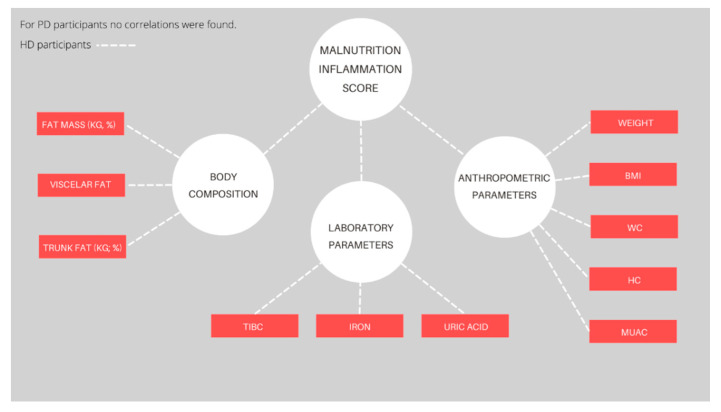
Significant correlations of the Malnutrition Inflammation Score and measured parameters for each dialysis modality. Abbreviations: BMI—Body Mass Index, WC—waist circumference, HC—hip circumference, MUAC—middle-upper arm circumference, TIBC—total iron-binding capacity. The red color depicts negative correlations (*p* < 0.05).

Figure 2 provides the correlation coefficients between MIS score and observed parameters (only statistically significant parameters are shown) for each dialysis modality. The detailed numerical data are shown in Table S3. For HD participants, negative correlations were found for MUAC (*p* = 0.02), WC (*p* = 0.02), HC (*p* = 0.03), weight (*p* < 0.001), BMI (*p* = 0.03), fat mass (%, *p* = 0.04 and kg, *p* = 0.01), visceral fat (*p* < 0.001), trunk fat mass (%, *p* = 0.01 and kg, *p* < 0.001), iron (*p* = 0.04), TIBC (*p* = 0.02), and uric acid (*p* = 0.02). Positive correlations were not determined for any of the observed parameters. Furthermore, neither positive nor negative correlations were found for PD participants.

**Figure 3:**
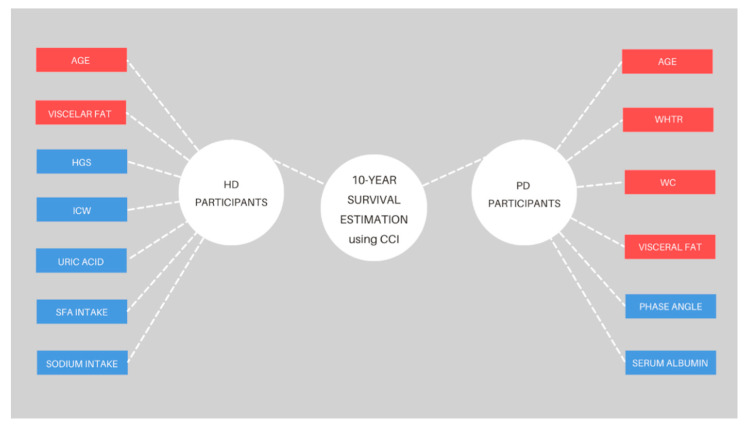
Significant correlations of the 10-year survival estimation and measured parameters for each dialysis modality. Abbreviations: CCI—Charlson Comorbidity Index, HGS—handgrip strength, ICW—intracellular water, SFA—saturated fatty acid, WHtR—waist-to-height ratio, WC—waist circumference. The red color depicts negative correlations and the blue color depicts positive correlations (*p* < 0.05).

Figure 3 provides the correlation coefficients between the estimated 10-year survival and observed parameters (only statistically significant parameters are shown). For HD participants, negative correlations were found for age (*p* < 0.001) and visceral fat level (*p* = 0.01), whereas positive correlations were found for handgrip strength (*p* = 0.04), ICW (*p* = 0.04), uric acid (*p* < 0.001), saturated fatty acids (*p* = 0.02), and sodium (*p* = 0.03) intake. For PD participants, negative correlations were found for age (*p* < 0.001), WC (*p* = 0.03), WHtR (*p* = 0.02), and visceral fat level (*p* < 0.001). Positive correlations were found for phase angle (*p* = 0.01) and serum albumin levels (*p* = 0.02).

## 4. Discussion

The routine assessment of nutritional status in patients undergoing dialysis, which includes an evaluation of body composition, muscle function, dietary intake, and laboratory parameters, remains a challenge due to a lack of time and structured nutritional care. Therefore, the main objective of this study, conducted with 55 participants undergoing HD, and 20 participants undergoing PD, was to analyze the differences in nutritional status and dietary intake in Dalmatian patients treated with PD and HD, and to determine adherence to the dietary recommendations specific for this population of patients. To the best of our knowledge, this is the first study to assess the differences in nutritional and hydration status and dietary intake in HD and PD patients in our region. 

As presented in the results, HD participants were older and had more comorbidities, such as diabetes mellitus and malignant diseases, than PD participants. The age difference in terms of dialysis modality was expected, since continuous ambulatory PD depends on patients’ cooperation [31], but also offers a better quality of life that allows them to remain flexible and keep their jobs [32,33], thus favoring more young and active patients. Although the incidence of diabetes mellitus onset is increasing with age [34] and, given the age difference, is more likely present in HD participants, diabetic patients are less likely to be treated with PD, mainly because of the fluctuations in glycemic control due to dialysate glucose absorption, a higher prevalence of PD-associated peritonitis, a rapid deterioration of kidney function due to inflammation and proteinuria, diabetic complications such as visual impairments and peripheral neuropathy, as well as overhydration [35,36]. Moreover, advancing age is one of the major factors for malignancy [37]. In addition, the decline in kidney function is associated with an increased risk of malignancy. The risk itself increases by 29% with each decrease in the estimated glomerular filtration rate (eGFR) of 10 mL/min, with the greatest risk observed at an eGFR lower than 40 mL/min [38,39]. Contrarily, Lee et al. did not find a significant difference for cancer risk between HD and PD participants that were age- and sex-matched [40].

Regarding body composition and anthropometric parameters, PD participants were overweight and had significantly higher fat mass and trunk fat mass when compared to the HD participants. Similar results were noticed in a Taiwanese study where PD participants had higher BMI and fat values, but lower lean tissue mass [41], and in a Korean study, where higher mean BMI and visceral fat were observed in PD participants [42]. Contrary to our results, Van Biesen et al. observed an equal BMI and fat tissue index among matched European HD and PD participants, with a higher lean tissue index in PD participants [43]. In a Spanish study by Di-Gioia et al., no differences in body composition were found [44]. The data on body composition and BMI with respect to dialysis modality are quite inconsistent, and the reason for the resulting discrepancies could be in the dialysis vintage itself [45]. According to a recent study conducted with 359 Caucasians undergoing dialysis, PD participants with a shorter dialysis vintage had higher lean tissue mass, whereas the fat mass and BMI were comparable between HD and PD participants. On the other hand, participants on long-term HD had higher lean tissue mass and lower fat mass values than the corresponding PD participants [45]. Different dialysis modalities have been found to have different effects on fluid volume control. Considering continuous ultrafiltration, as well as the fact that residual renal function is better preserved in PD patients, it is expected that PD treatment should ensure better fluid volume control [46]. In the present study, there were no significant differences in hydration parameters between HD and PD participants. Similarly, in the study by van der Sande et al., fluid status was comparable between PD participants and HD participants when predialytic measurements were considered, whereas lower fluid levels were noticed in HD participants after a dialysis session [47]. The same results were obtained in a Belgian study [48] with 44 HD and 34 PD participants, as well as a Turkish study with 43 HD and 33 PD participants [46], suggesting that overhydration might be a more frequent and severe problem in PD than HD patients. The reason for this could be the more effective control of extracellular volume overload, and also, the more frequent assessment of fluid status in HD participants in comparison to PD participants [46]. Overhydration or fluid overload is a common complication in dialysis patients, and has been associated with a variety of outcomes, including hypertension, arterial stiffness, left ventricular hypertrophy, atherosclerosis, uremic cardiomyopathy, and cardiovascular morbidity and all-cause mortality, making an adequate assessment of fluid status in this patient population of paramount importance [49]. Furthermore, the risk of fluid overload increases with a more severe depletion of lean tissue or adipose tissue, and further worsens when inflammation is present [50].

In contrast to most other studies, in which there was no difference in HGS related to dialysis modality [51,52], we observed lower HGS in HD participants. The reasons for the disparity in the results could be the age and body composition of the participants. In general, factors such as age, gender, and body mass [53] could influence the HGS, with higher values observed in individuals aged 30 to 45 years, overweight and obese individuals, as well as males [54]. Furthermore, HGS values were associated with age and gender in patients undergoing maintenance dialysis [54]. Similar results were noticed in a Korean study that included 93 HD participants and 67 PD participants [55].

Higher serum albumin, glucose, and potassium levels in HD participants and higher total cholesterol and LDL cholesterol levels in PD participants were observed in the present study. A decrease in serum albumin levels for each 10 g/L is associated with increased mortality risk in HD and PD patients [56], whereas serum albumin levels lower than 38 g/L are associated with a significant increase in death rates among PD patients [57]. One of the major disadvantages of the PD, when compared to HD, is protein loss through peritoneal effluent, with an average albumin loss of 4 g per 24 h [58]; therefore, PD patients are at a higher risk for hypoalbuminemia. These losses are usually exceeded by the albumin synthesis in the liver, but the rate of synthesis could be suppressed due to inflammation and malnutrition [58]. Considering the higher prevalence of diabetes mellitus in HD participants, higher glucose levels were expected in this group of participants. It has been demonstrated that patients treated with PD have a lower risk of developing hyperkalemia in comparison to HD patients [59] due to the continuous nature of PD treatment [60], the retained residual renal function being longer than HD patients, and the higher usage of diuretics that increase the urinary secretion of potassium cations [61]. The lipid profile of PD patients differs from that of HD patients [62]. Higher total cholesterol and LDL cholesterol levels found in PD patients [63,64] might be related to glucose absorption, peritoneal protein loss, and decline in residual kidney function [65,66,67]. Paradoxically, elevated TC/HDL cholesterol levels in patients treated with HD are thought to play a protective role, being associated with lower mortality rates [68]. In contrast, a recent prospective study found that higher TC/HDL cholesterol levels in PD patients might be a risk factor for mortality, which is consistent with the general population [69]. Mandatory glucose absorption has been shown to be associated with several unfavorable metabolic complications, such as hyperglycemia, increased insulin need, weight gain and increased visceral fat, dyslipidemia, and metabolic syndrome [70]. The negative effects of excessive glucose and glucose degradation product exposure can be reduced by using low glucose degradation products solutions, as well as non-glucose solutions, such as amino acids, icodextrin, or their combination [71].

PD participants in the present study had a higher intake of several nutrients, whereas the overall dietary intake, shown as MAR, did not differ between the observed groups of participants. The higher energy intake in PD participants can be explained by an additional energy intake of about 400 kcal from mandatory peritoneal glucose absorption [55]. Contrary to the above-mentioned results, a few studies that compared dietary intake based on the dialysis modality using a food diary and 24-h dietary recall reported no significant differences in energy intake, even when including PD dialysate calories [72,73,74]. Higher energy intake in HD patients was observed in the study, in which dietary intake was assessed using semi-FFQ. The discrepancy in the results could arise from the applied method, given that the FFQ is intended for use on a larger number of participants, and that dietary intake assessment is often underestimated or overestimated due to the limited number of included foods that an individual can consume [55]. In addition, FFQ is not sensitive to nutrient loss due to thermic processing [18]. Protein intake did not differ between PD and HD participants, which is in line with the results from a study by Johansson et al. [74]. On the other hand, Harvinder et al., as well as Kim et al., found that HD patients had significantly higher protein intake in comparison with PD participants [55,72]. Chronic inflammation, the loss of residual renal function [75], and the additional protein loss to the peritoneal fluid [76] can contribute to inadequate protein intake in these patients. The results considering micronutrient intake were somewhat expected, considering that PD is performed daily. Therefore, the body does not accumulate as much potassium, sodium, and phosphorus, and the diet is often more generous compared to patients treated with HD [55]. Furthermore, the results from the present study showed insufficient overall dietary intake (MAR) when compared with recommended intake characteristics for end-stage renal disease patients, without significant differences between HD and PD participants. When observing each micronutrient, the intake over the recommended allowance was noticed only for thiamin and niacin in both groups of participants, and, additionally, phosphorus for PD participants. A significant difference in the adequacy of intake for potassium and phosphorus was observed between HD and PD participants. Inadequate dietary intake when compared to the dietary recommendations for dialysis patients was reported in most of the studies [55,73,77,78], indicating that an unbalanced diet is one of the main risk factors for malnutrition in this specific population of patients. All of these results highlight the difficulties that dialysis patients and their families encounter daily when planning and preparing kidney-friendly meals.

MIS values of less than 6, indicating no malnutrition to mild malnutrition, were seen in 53% of HD participants and 55% of PD participants. Differences between the HD and PD groups of participants were noticed for serum albumin and TIBC levels and the number of comorbidities, including the number of years spent on dialysis, but not for an overall score. Different percentages of malnutrition were reported, as defined by MIS. Naini et al. compared the degree of malnutrition between HD and PD patients without differences in dialysis modality, but found a higher number of patients with no-to-mild malnutrition (79.6% and 72.7% in PD and HD patients, respectively) than in the present study [79]. On the other hand, Naeeni et al. reported that 90.3% of the PD patients had no or mild malnutrition [80]. Similar to our results, the mean MIS of HD patients in other studies varied from 4 to 6 [81,82,83]. One of the major drawbacks of MIS and the reason for the discrepancies between these studies is the lack of a specified cutoff point. Furthermore, dialysis vintage, which correlates positively with the values of MIS, may lead to inflammation, nutrient loss, and hypercatabolism. Regarding the individual MIS variables, similar results were found in a Greek study which included 47 HD participants and 27 PD participants [83]. In the present study, MIS negatively correlated with anthropometric and body composition parameters, such as fat mass and fat mass percentage, visceral fat level, trunk fat mass, and trunk fat percentage, in HD participants. Most of the studies reported a negative correlation with body weight, BMI, and MUAC [26,84]. Regarding biochemical parameters, TIBC, iron, and uric acid negatively correlated with MIS, which is in line with the results demonstrated in other studies [26,85]. Furthermore, a negative TIBC correlation was logical and expected, as TIBC is a part of the questionnaire. No correlations between MIS and observed parameters were found for PD participants. A possible explanation for these findings could be due to a relatively small number of PD participants included in the present study.

The estimated 10-year survival, calculated using CCI, negatively correlated with visceral fat in HD participants, and visceral fat, WC, and WHtR in PD participants. This is in line with results from other studies, considering that visceral fat is associated with inflammation and metabolic abnormalities, and as such, is a risk factor for CVD and mortality in this specific population of patients [86,87]. Furthermore, Castro et al. demonstrated that high WC and an increase in WC over time were predictors of mortality in PD patients [88]. Similar to our results, Vogt et al. showed that HGS was associated with mortality independent of dialysis modality [51]. Intracellular water positively correlated with estimated survival in the HD participants in the present study. A study conducted with 124 HD patients showed that ICW was an independent risk factor for mortality, and was correlated with lower muscle mass and a higher inflammation rate [89]. In line with our findings, Huang et al. showed that a lower phase angle is a marker for increased mortality rate in PD patients [90]. Regarding laboratory parameters, estimated 10-year survival positively correlated with uric acid in HD participants, and serum albumin in PD participants. The results from the previous studies suggest that a lower uric acid level is associated with a higher risk of all-cause mortality among HD patients [91]. Low salt intake is associated with all-cause mortality in HD patients, which could be due to malnutrition resulting from the excessive salt reduction in the diet [92]. The intake of saturated fatty acids positively correlated with an estimated 10-year survival, which is contradictory considering the well-known negative effects of saturated fatty acids on cardiovascular health [93].

This research has a few limitations. Due to the cross-sectional design, no causal relations could be determined. The difference in age, duration of dialysis, and comorbidities between HD and PD participants is considered a limitation of the study due to the direct influence of the mentioned parameters on nutritional status. According to the KIDGO guidelines, it is necessary to perform a food diary or some other method of assessing dietary intake for at least 3 days, including days spent on dialysis. Furthermore, a possible limitation could be using approximation of glucose absorption through peritoneal membrane instead of calculating glucose absorption according to the different PD fluid prescribed to each PD participant included in this study. Additionally, data about supplementation and pharmacological therapy are lacking. Finally, a relatively small number of participants from a single center were included.

## 5. Conclusions

The results from this study showed that the prevalence of malnutrition, as defined by the MIS, is high among Dalmatian HD and PD participants. Therefore, the dietary intake for both HD and PD participants, as determined by 24-h dietary recalls, did not meet the current dietary recommendations for this specific population. Additionally, a significant difference in fat tissue parameters was found between HD and PD participants, whereas significant differences in hydration status and muscle mass parameters were not found. The estimated 10-year survival, on average, was 2% for HD participants and 53% for PD participants, and it correlated with several parameters of nutritional status in HD and PD participants, as well as nutrient intake in HD participants. These results indicate the need for regular nutritional assessments and individualized nutritional care to ensure patient education, and consistent, appropriate nutritional intake in order to improve the nutritional status and quality of life in this population of patients, as well as reduce their mortality. 

## Figures and Tables

**Table 1:** Basic characteristics and differences regarding dialysis modality.

	HD (*N* = 55)	PD (*N* = 20)	*p* *
Basic characteristics			
Sex (female), *N* (%)	17 (30.9)	9 (45)	0.26
Age (years), median (IQR)	69 (56–76)	53.5 (36.25–66.25)	0.002
Dialysis duration (months), median (IQR)	47 (22–77)	24 (8.5–36)	0.001
Presence of AH, *N* (%)	35 (78)	17 (85)	0.50
Presence of DM, *N* (%)	15 (32)	1 (5)	0.03
Presence of MD, *N* (%)	14 (31)	0	0.003
Charlson Comorbidity Index, median (IQR)	6 (4–8)	4 (2–6)	<0.001
Estimated 10-year survival (%), median (IQR)	2 (0–53)	53 (15–87)	<0.001
Anthropometric parameters			
Weight (kg), median (IQR)	73.5 (66.4–83.5)	80.75 (70.53–101.7)	0.06
Height (cm), median (IQR)	175 (164–183)	173 (171–178.75)	0.90
BMI (kg/m^2^), median (IQR)	24.6 (22.2–27.6)	25.65 (23.53–31.38)	0.10
Middle upper arm circumference (cm), median (IQR)	28 (26.25–31.25)	30.25 (26.63–33.75)	0.23
Waist circumference (cm), median (IQR)	94 (89.5–101.75)	98.5 (90.25–103.75)	0.25
Hip circumference (cm), median (IQR)	102 (98.5–108.25)	108.5 (97.5–114.5)	0.06
WHR, median (IQR)	0.92 (0.87–0.98)	0.91 (0.85–0.93)	0.32
WHtR, median (IQR)	0.54 (0.51–0.59)	0.56 (0.52–0.61)	0.40
Handgrip strength (kg), median (IQR)	25.7 (17.7–32.3)	31.3 (25.4–41.58)	0.01
Body composition			
Total body water (kg), median (IQR)	43.3 (36.6–50.6)	42.15 (38.13–48.13)	0.98
Extracellular water (kg), median (IQR)	18.2 (16.4–20.7)	19.55 (17.28–20.88)	0.34
Intracellular water (kg), median (IQR)	25.1 (20.7–29.6)	23.65 (20.25–28.1)	0.59
Fat mass (kg), median (IQR)	14.4 (7.1–20.4)	19.25 (12.1–29.98)	0.01
Fat mass (%), median (IQR)	18.5 (11.3–25.6)	24.5 (16.68–33.2)	0.02
Fat-free mass (kg), median (IQR)	61.9 (53.3–70.1)	62.2 (55.3–69.38)	0.66
Visceral fat level, median (IQR)	9 (8–11)	8 (4.5–13.5)	0.34
Muscle mass (%), median (IQR)	58.8 (50.6–66.6)	59.1 (52.48–65.98)	0.66
Phase angle (°), median (IQR)	4.8 (4.3–5.8)	5.2 (4.73–5.88)	0.07
Trunk fat mass (kg), median (IQR)	7.1 (3.4–11.55)	9.6 (6.65–17.75)	0.02
Trunk fat mass (%), median (IQR)	16.9 (9.4–24)	22.3 (16.15–31.35)	0.01
SMI, median (IQR)	7.93 (7.13–9.11)	8.59 (7.6–9.37)	0.27

* *p*-values were obtained with the Chi-squared Test for categorical data and the Mann–Whitney U test for non-parametric numerical data (*p* < 0.05). Abbreviations: *N*—number, IQR—interquartile range, AH—arterial hypertension, DM—diabetes mellitus, MD—malignant disease, BMI—Body Mass Index (kg/m^2^), WHR—waist-to-hip ratio, WHtR—waist-to-height ratio, SMI—sarcopenic muscle index.

**Table 2:** Biochemical parameters and differences regarding dialysis modality.

	HD (*N* = 55) Median (IQR)	PD (*N* = 20) Median (IQR)	*p* *
Hb (g/L)	116 (111–122)	115.5 (100.75–122.75)	0.82
MCV (fL)	93.9 (89.25–98.05)	88.75 (87–93.38)	0.03
Iron (µmol/L)	12 (10–14)	14 (10–18)	0.43
TIBC (µmol/L)	40 (35.5–46)	44 (39.75–52.5)	0.02
FBG (mmol/L)	6.1 (4.95–7.45)	5.55 (4.88–6)	0.04
Uric acid (µmol/L)	335 (294–370)	329.5 (279.5–362.5)	0.53
Total cholesterol (mmol/L)	3.8 (3–4.4)	4.45 (3.63–6.83)	0.04
Triglycerides (mmol/L)	1.7 (1.1–2.3)	1.8 (0.8–4.28)	0.68
HDL cholesterol (mmol/L)	1 (0.9–1.3)	1.15 (0.88–1.3)	0.66
LDL cholesterol (mmol/L)	1.8 (1.2–2.6)	2.7 (2–3.48)	0.04
Serum albumin (g/L)	41.8 (39.9–43.65)	38.1 (35.8–40.53)	<0.001
Sodium (mmol/L)	137 (135–139)	139 (134.25–140)	0.49
Potassium (mmol/L)	5.5 (5–5.95)	4.3 (4.13–4.88)	<0.001
Chloride (mmol/L)	99 (97.25–101)	97 (95–101)	0.09
Calcium (mmol/L)	2.23 (2.16–2.3)	2.23 (2.14–2.41)	0.96
Phosphate (mmol/L)	1.7 (1.31–2.07)	1.75 (1.51–1.89)	0.47
Magnesium (mmol/L)	1.04 (0.98–1.15)	1.03 (0.65–1.22)	0.75
CRP (mg/L)	3.4 (1.3–9.35)	2.6 (1.78–10.15)	0.77
iPTH (pmol/L)	27.58 (16.11–48.58)	23.2 (6.95–90.33)	0.89

* *p*-values were obtained with the Mann–Whitney U test (*p* < 0.05). Abbreviations: *N*—number, IQR—interquartile range, Hb—hemoglobin, MCV—Mean Corpuscular Volume, TIBC—total iron-binding capacity, FBG—fasting blood glucose, HDL—high-density lipoproteins, LDL—low-density lipoproteins, CRP—*C*-reactive protein, iPTH—intact parathyroid hormone.

**Table 3:** Dietary intake and differences regarding dialysis modality.

	HD (*N* = 55)	PD (*N* = 20)	*p* *
24-h dietary recall			
Energy (kcal)	1321.8 (1038–1608.6)	1890.1 (1651.55–2300.05)	<0.001
Protein (g)	63.7 (43–77.5)	70.25 (48.05–103.58)	0.14
Fat (g)	52.9 (36.6–70.3)	82.5 (52.73–89.18)	0.008
SFA (g)	19.2 (12.8–27.9)	25.8 (19.75–38.95)	0.02
MUFA (g)	15.4 (11.7–22.3)	24.45 (15.45–30.4)	0.05
PUFA (g)	7 (5–8.9)	8.4 (4.83–12.6)	0.20
Cholesterol (mg)	185.8 (111.2–279)	210.45 (146.5–262.5)	0.39
Carbohydrates (g)	140.7 (111–186.9)	148.7 (121.75–164.65)	0.76
Dietary fibres (g)	10.9 (7.8–16.1)	15.3 (10.93–18.28)	0.04
Vitamin A (IU)	318 (246–443.2)	419.5 (268.18–729.48)	0.08
Vitamin D (μg)	2.1 (0.6–2.8)	1.7 (0.55–2.83)	0.94
Vitamin E (mg)	4.9 (3.2–8.1)	6.5 (3.73–11.45)	0.05
Vitamin K (μg)	31.5 (17.4–57.2)	39.25 (25.7–60.65)	0.20
Thiamine (mg)	1.2 (0.88–1.7)	1.35 (0.9–1.88)	0.47
Riboflavin (mg)	1.3 (0.8–1.7)	1.35 (0.9–2.2)	0.41
Niacin (mg)	20.5 (12.4–27.4)	23.3 (16.2–33.38)	0.15
Pantotenic acid (mg)	3.5 (2.5–4.8)	3.9 (2.53–6)	0.43
Vitamin B6 (mg)	1.1 (0.7–1.8)	1.45 (1.03–2.03)	0.16
Folic acid (μg)	130.2 (102.4–218.5)	164.55 (121.68–235.58)	0.17
Cholin (mg)	210.6 (120.4–267.9)	254.45 (145.73–317.93)	0.28
Vitamin B12 (μg)	2.5 (1.5–4.3)	3.2 (1.55–4.83)	0.37
Vitamin C (mg)	36.2 (14.2–68.8)	48.2 (20.3–75.55)	0.43
Calcium (mg)	625.8 (362.9–839.7)	698.45 (497.95–1012.8)	0.18
Iron (mg)	10.5 (7.2–13.4)	11.1 (7.58–12.08)	0.59
Magnesium (mg)	151.9 (132–207.6)	217.5 (165.1–265.65)	0.02
Phosphate (mg)	799.5 (522–973.7)	939.1 (693.28–1281.05)	0.04
Zinc (mg)	7.1 (4.5–9.6)	7.6 (5.93–12.88)	0.14
Copper (mg)	0.7 (0.5–1)	1.1 (0.8–1.38)	0.005
Potassium (mg)	1597.3 (1162.3–2026.3)	2168.7 (1621.28–2734.45)	0.02
Sodium (mg)	1888.8 (1240.2–2457.1)	1993 (1598.03–3143.53)	0.18

* *p*-values were obtained with the Mann–Whitney U test. Abbreviations: *N*—number, SFA—saturated fatty acids, MUFA—monounsaturated fatty acids, PUFA—polyunsaturated fatty acids.

**Table 4:** Malnutrition Inflammation Score and differences regarding dialysis modality.

	HD (*N* = 55)	PD (*N* = 20)	*p* *
Dry mass change			
No change	32 (58)	14 (70)	0.82
Minor weight loss (0.6 kg–1 kg)	8 (15)	1 (5)	
Weight loss more than 1 kg but <5%	9 (16)	3 (15)	
Weight loss > 5%	5 (9)	2 (10)	
Dietary intake			
Good appetite and no deterioriation of dietary intake	47 (85)	16 (80)	0.72
Somewhat sub-optimal solid diet intake	8 (15)	4 (20)	
Gastrointestinal symptoms			
No symptoms with good appetite	40 (73)	13 (65)	0.81
Mild symptoms	13 (24)	6 (30)	
Frequent diarrhea or vomiting or severe anorexia	2 (4)	1 (5)	
Nutritionally related functional impairment			
Normal-to-improved functional capacity	24 (44)	13 (65)	0.29
Occasional difficulty with baseline ambulation	30 (55)	7 (35)	
Bed/chair-ridden or little-to-no physical activity	1 (2)	0	
Co-morbidities including number of years on dialysis			
On dialysis less than one year and healthy otherwise	4 (7)	6 (30)	0.01
Dialyzed for 1–4 years or mild co-morbidity	23 (42)	11 (55)	
Dialyzed > 4 years or moderate co-morbidity	22 (40)	3 (15)	
Any severe multiple co-morbidity	6 (11)	0	
Decreased fat stores or loss of subcutaneous fat			
Normal (no change)	29 (53)	12 (60)	0.92
Mild	22 (40)	7 (35)	
Severe	4 (7)	1 (5)	
Signs of muscle wasting			
Normal (no change)	26 (47)	10 (50)	>0.99
Mild	26 (47)	9 (45)	
Severe	3 (5)	1 (5)	
Body mass index (BMI)			
BMI ≥ 20 kg/m^2^	53 (96)	20 (100)	>0.99
BMI 18–19.99 kg/m^2^	1 (2)	0	
BMI 16–17.99 kg/m^2^	1 (2)	0	
Serum albumin			
≥40 (g/L)	40 (73)	6 (30)	<0.001
35–39 (g/L)	15 (27)	11 (55)	
30–34 (g/L)	0	2 (10)	
≤30 (g/L)	0	1 (5)	
Serum TIBC (total iron-binding capacity)			
>44.75 µmol/L	18 (33)	14 (70)	0.006
35.8–44.6 µmol/L	23 (42)	6 (30)	
26.8–35.7 µmol/L	14 (25)	0	
MIS	5 (4–8)	5 (2–7)	0.19 ^†^
MIS < 6	29 (53)	11 (55)	0.93

* *p*-values were obtained with the Chi-squared test and Mann–Whitney U ^†^ (*p* < 0.05). Abbreviations: *N*—number, HD—hemodialysis, PD—peritoneal dialysis, MIS—Malnutrition Inflammation Score.

## Data Availability

Raw data can be found at the corresponding author via e-mail: josiparadic1973@gmail.com.

## Supplementary Materials

The following supporting information can be downloaded at: https://www.mdpi.com/article/10.3390/nu14173553/s1, Table S1. Lifestyle questionnaire and differences regarding dialysis modality, Table S2. Nutrient adequacy ratios and differences regarding dialysis modality Table S3. Significant correlations of Malnutrition Inflammation Score and measured parameters.
