# Supplementary material for: Nutritional and Hydration Status and Adherence to Dietary Recommendations in Dalmatian Dialysis Patients
Source: Nutrients. 2022 Aug 29;14(17):3553. doi: 10.3390/nu14173553 (PMC9460881; doi:10.3390/nu14173553)
Supplement: Supplementary file 1 [file nutrients-14-03553-s001.zip › nutrients-1885615-supplementary.pdf]

Supplementary materials

Table S1. Lifestyle questionnaire and differences regarding dialysis modality.

	HD (N=55)	PD (N=20)	*p**
**LIFSTYLE QUESTIONNAIRE, N (%)**			
Sex			
Male	38 (69)	11 (55)	0.26
Female	17 (31)	9 (45)	
Education level			
Elementary school	11 (20)	4 (20)	0.19
Middle school	34 (62)	10 (50)	
Intermediate education	4 (7)	5 (25)	
Faculty education or higher	6 (11)	1 (5)	
Working status			
Employed full-time	2 (4)	8 (40)	<0.001
Unemployed	4 (7)	2 (10)	
Student	0	1 (5)	
Retired	45 (82)	9 (45)	
Housewife	1 (1.8)	0	
Sick leave	3 (5.5)	0	
Inhabitant of			
Village	11 (20)	7 (35)	0.18
Town/city	44 (80)	13 (65)	
Monthly household budget			
< 2850 kn	8 (15)	1 (5)	0.42
2850 – 5700 kn	15 (27)	3 (16)	
5700 – 8600 kn	12 (22)	7 (37)	
8600 – 11400 kn	11 (20)	3 (16)	
> 11400 kn	9 (16.4)	5 (26.3)	
Number of people in a household			
1	10 (18)	2 (10)	0.11
2	21 (38)	8 (40)	
3	14 (25)	1 (5)	
4	4 (7)	4 (20)	
5	3 (5.5)	3 (15)	
6	2 (3.6)	2 (10)	
9	1 (2)	0	
Physical activity intensity			
Extremely weak	1 (2)	3 (15)	0.10
Weak	22 (40)	9 (45)	
Moderate	29 (53)	8 (40)	
Intense	3 (5)	0	
Liquid intake			
Does not consume liquid	1 (2)	0	0.50
1 glass	14 (25)	3 (16)	
2 glasses	14 (25)	5 (26)	
3 glasses	15 (27)	3 (16)	
4 glasses	7 (12.7)	5 (26.3)	
5 glasses	4 (7.3)	3 (15.8)	
Loss of appetite, yes	10 (18)	1 (5)	0.27

Regular stool	41 (75)	17 (85)	0.53
Nausea	15 (27)	1 (5)	0.05
Have chewing/swallowing problem	7 (13)	1 (5)	0.67
Taking dietary supplements	28 (51)	9 (45)	0.80
Taking ONS	24 (44)	9 (45)	> 0.99
Taking ONS as prescribed by a specialist	17 (71)	5 (56)	0.44
Number of meals			
1	1 (2)	0	0.64
2	7 (13)	1 (5)	
3	19 (35)	8 (40)	
4	24 (44)	9 (45)	
5	4 (7.3)	1 (5)	
6	0	1 (5)	

**p*-values were obtained with Chi-square test. Abbreviations: HD- hemodialysis, PD- peritoneal dialysis, ONS- oral nutritional support.

Table S2. Nutrient adequacy ratios and differences regarding dialysis modality.

	NUTRIENT ADEQUACY<br>Median (IQR)			
NAR energy	0.55 (0.45-0.66)	0.88 (0.76-0.92)	0.64 (0.49-0.83)	0.07
NAR protein	0.65 (0.46-0.81)	0.66 (0.46-0.8)	0.65 (0.47-0.81)	0.48
NAR Na	0.82 (0.54 - 1.07)	0.87 (0.69 - 1.37)	0.84 (0.62 - 1.15)	0.18
NAR K	0.59 (0.43 - 0.74)	0.8 (0.6 - 1.01)	0.63 (0.45 - 0.85)	0.01
NAR Ca	0.78 (0.45 - 1.05)	0.88 (0.62 - 1.27)	0.79 (0.47 - 1.09)	0.17
NAR P	0.8 (0.52 - 0.97)	1.18 (0.87 - 1.61)	0.86 (0.6 - 1.07)	<0.001
NAR Vitamin C	0.33 (0.14 - 0.63)	0.44 (0.2 - 0.71)	0.34 (0.15 - 0.67)	0.38
NAR Vitamin D	0.14 (0.04 - 0.19)	0.12 (0.04 - 0.19)	0.13 (0.04 - 0.19)	0.96
NAR Thiamin	12 (8.78 - 17)	13.5 (9 - 18.75)	12 (9 - 17)	0.47
NAR Riboflavin	0.81 (0.5 - 1.06)	0.85 (0.56 - 1.38)	0.81 (0.5 - 1.13)	0.41
NAR Niacin	12.81 (7.75 - 17.13)	14.57 (10.13 - 20.86)	13.38 (9.13 - 17.94)	0.15
NAR B6	0.69 (0.44 - 1.12)	0.88 (0.63 - 1.21)	0.77 (0.47 - 1.13)	0.17
NAR Folic acid	0.39 (0.31 - 0.66)	0.5 (0.37 - 0.71)	0.45 (0.32 - 0.68)	0.16
NAR B12	0.63 (0.38 - 1.08)	0.8 (0.39 - 1.21)	0.65 (0.38 - 1.13)	0.37
MAR	0.68 (0.52 – 0.74)	0.79 (0.65 – 0.82)	0.69 (0.63 – 0.78)	0.20

p-values were obtained with the Mann-Whitney U test for non-parametric numerical data. Abbreviations: IQR- interquartile range, SFA- short-chain fatty acid, MUFA- monounsaturated fatty acid, PUFA- polyunsaturated fatty acid, NAR- nutrient adequacy ratio, MAR- mean adequacy ratio, Na- sodium, K- potassium, Ca- calcium, P- phosphate.

Table S3. Correlations of Malnutrition Inflammation Score and measured parameters.

	HD (N=55)	PD (N=20)
**Parameters**	**R (*p*-value)**	**R (*p*-value)**
Middle upper arm circumference (cm)	-0.331 (0.02)	-0.266 (0.26)
Waist circumference (cm)	-0.317 (0.02)	-0.358 (0.12)
Hip circumference (cm)	-0.297 (0.03)	-0.318 (0.17)
Weight (kg)	-0.387 (<0.001)	-0.355 (0.12)
BMI (kg/m²)	-0.290 (0.03)	-0.333 (0.15)
TBW (kg)	-0.193 (0.16)	-0.264 (0.26)
ECW (kg)	-0.245 (0.07)	-0.33 (0.16)
ICW (kg)	-0.144 (0.29)	-0.224 (0.34)
Fat mass (%)	-0.282 (0.04)	-0.173 (0.47)
Fat mass (kg)	-0.368 (0.01)	-0.273 (0.24)
Visceral fat	-0.386 (<0.001)	-0.202 (0.39)
Fat-free mass (kg)	-0.213 (0.12)	-0.412 (0.07)
Muscle mass (%)	-0.213 (0.12)	-0.412 (0.07)
Trunk fat (%)	-0.378 (0.01)	-0.265 (0.26)
Trunk fat (kg)	-0.415 (<0.001)	-0.389 (0.09)
TIBC (μmol/L)	-0.288 (0.04)	0.217 (0.52)
Uric acid (mmol/L)	-0.332 (0.02)	-0.655 (0.2)
Triglycerides (mmol/L)	-0.32 (0.02)	-0.18 (0.47)

p-values were obtained with the Spearman´s coefficient of rank correlation Rho. Abbreviations: N- number, BMI-Body mass index, TBW-Total body water (kg), ECW-Extracellular water (kg), ICW-Intracellular water (kg), TIBC-total iron-binding capacity.
